# *Ex vivo *activity of the ACT new components pyronaridine and piperaquine in comparison with conventional ACT drugs against isolates of *Plasmodium falciparum*

**DOI:** 10.1186/1475-2875-11-45

**Published:** 2012-02-14

**Authors:** Aurélie Pascual, Philippe Parola, Françoise Benoit-Vical, Fabrice Simon, Denis Malvy, Stéphane Picot, Pascal Delaunay, Didier Basset, Danièle Maubon, Bernard Faugère, Guillaume Ménard, Nathalie Bourgeois, Claude Oeuvray, Eric Didillon, Christophe Rogier, Bruno Pradines

**Affiliations:** 1 Unité de Recherche en Biologie et Epidémiologie Parasitaires Unité de Recherche pour les Maladies Infectieuses et Tropicales Emergentes UMR--6236, Institut de Recherche Biomédicale des Armées, Allée du Médecin-colonel Jamot,-BP 60109, 13262 Marseille Cedex, France; 2 Centre National de Référence du Paludisme, Marseille, France; 3 Institut Hospitalo-Universitaire en Maladies Infectieuses et Tropicales, Hôpital Nord, Marseille, France; 4 Laboratoire de Parasitologie-Mycologie, Centre Hospitalier Universitaire de Rangueil, Toulouse, France; 5 Service de Pathologie Infectieuse et Tropicale, Hôpital d'Instruction des Armées Laveran, Marseille, Frnace; 6 Travel Clinics and Division of Tropical Medicine and Imported Diseases, Centre Hospitalier Universitaire, Bordeaux, France; 7 Malaria Research Unit, UMR 5246, CNRS Lyon, France; 8 Laboratoire de Parasitologie-Mycologie, Hopital de 1'Archet, Nice, France; 9 Laboratoire de Parasitologie-Mycologie, Centre Hospitalier Universitaire Lapeyronnie, Montpellier, France; 10 Laboratoire de Parasitologie-Mycologie, Centre Hospitalier Universitaire et Université de Grenoble 1, Grenoble, France; 11 Laboratoire de Parasitologie-Mycologie, Centre Hospitalier Universitaire La Timone, Marseille, France; 12 Fédération des Laboratoires, Hôpital d'Instruction des Armées Sainte Anne, Toulon, France; 13 Service de Bactériologie-Virologie-Parasitologie, Centre Hospitalier Universitaire Caremeau, Nimes, France; 14 Medicines for Malaria Venture, Geneva, Switzerland; 15 Fulcrum Pharma (Europe) Ltd, Hemel Hempstead, UK

**Keywords:** Malaria, *Plasmodium falciparum*, Anti-malarial, *In vitro*, Resistance, Pyronaridine, Piperaquine

## Abstract

**Background:**

The aim of the present work was to assess i) *ex vivo *activity of pyronaridine (PND) and piperaquine (PPQ), as new components of artemisinin-based combination therapy (ACT), to define susceptibility baseline, ii) their activities compared to other partner drugs, namely monodesethylamodiaquine (MDAQ), lumefantrine (LMF), mefloquine (MQ), artesunate (AS) and dihydroartemisinin (DHA) against 181 *Plasmodium falciparum *isolates from African countries, India and Thailand, and iii) *in vitro *cross-resistance with other quinoline drugs, chloroquine (CQ) or quinine (QN).

**Methods:**

The susceptibility of the 181 *P. falciparum *isolates to the nine anti-malarial drugs was assessed using the standard 42-hours ^3^H-hypoxanthine uptake inhibition method.

**Results:**

The IC_50 _values for PND ranged from 0.55 to 80.0 nM (geometric mean = 19.9 nM) and from 11.8 to 217.3 nM for PPQ (geometric mean = 66.8 nM). A significant positive correlation was shown between responses to PPQ and PND responses (*rho *= 0.46) and between PPQ and MDAQ (*rho *= 0.30). No significant correlation was shown between PPQ IC_50 _and responses to other anti-malarial drugs. A significant positive correlation was shown between responses to PND and MDAQ (*rho *= 0.37), PND and LMF (*rho *= 0.28), PND and QN (*rho *= 0.24), PND and AS (*rho *= 0.19), PND and DHA (*rho *= 0.18) and PND and CQ (*rho *= 0.16). All these coefficients of correlation are too low to suggest cross-resistance between PPQ or PND and the other drugs.

**Conclusions:**

In this study, the excellent anti-malarial activity of PPQ and PND was confirmed. The absence of cross-resistance with quinolines and artemisinin derivatives is consistent with the efficacy of the combinations of PPQ and DHA or PND and AS in areas where parasites are resistant to conventional anti-malarial drugs.

## Background

During the past 20 years, many strains of *Plasmodium falciparum *have become resistant to chloroquine and other anti-malarial drugs [1]. This has prompted a search for an effective alternative anti-malarial drug with minimal side effects. The emergence and spread of parasites resistant to anti-malarial drugs has caused an urgent need for novel compounds to be discovered and developed. One strategy for reducing the prevalence of malaria is the combinatorial use of drugs. The combination protects each drug from the development of resistance and reduces the overall transmission of malaria [2]. The official first-line anti-malarial policy is now artemisinin-based combination therapy (ACT) [3]. The artemisinin derivative causes rapid and effective reduction of the parasite biomass and gametocytes carriage, while the partner drug, which has a longer duration of action, achieves effective clinical and parasitological cure. Different formulations of ACT were evaluated: artesunate-sulphadoxine-pyrimethamine [4], artesunate-amodiaquine [5], artemether-lumefantrine [6], artesunate-mefloquine [7], artesunate-chlorproguanil-dapsone [8], dihydroartemisinin-piperaquine [9] and artesunate-pyronaridine [10].

However, suspected decreased susceptibility of ACT or, at least, longer parasite clearance times have been described in Cambodia [11-14]. In addition, prior therapy by amodiaquine-containing ACT selected reduced response to monodesethylamodiaquine, suggested that amodiaquine-containing regimens may rapidly lose efficacy in Africa [15]. This emergence of parasite resistance to ACT indicates that novel compounds and combinations need to be discovered and developed.

The aim of the present work was to assess i) *ex vivo *activity of two recent ACT partner drugs, pyronaridine (PND) and piperaquine (PPQ) to define the susceptibility baseline, ii) comparison with standard components of ACT, such as monodesethylamodiaquine (the active metabolite of amodiaquine) (MDAQ), lumefantrine (LMF), mefloquine (MQ), artesunate (AS) or dihydroartemisinin (DHA), and iii) *in vitro *cross-resistance with other quinoline drugs, such as chloroquine (CQ) and quinine (QN).

## Methods

### Plasmodium falciparum isolates

In total, 181 *P. falciparum *isolates were collected between April 2008 and April 2010 from patients hospitalized in France with imported malaria from a malaria-endemic country (Angola, Benin, Burkina Faso, Cameroon, Comoros, Congo, Ivory Coast, Gabon, Gambia, Ghana, Guinea, India, Madagascar, Mali, Mozambique, Niger, Central African Republic, Senegal, Thailand, Togo, Zambia). Informed consent was not required for this study as the sampling procedures and testing are part of the French national recommendations for the care and surveillance of malaria. Venous blood samples were collected in Vacutainer^® ^ACD tubes (Becton Dickinson, Rutherford, NJ, USA) before treatment and transported at 4°C from French hospitals located in Marseille, Toulouse, Bordeaux, Lyon, Montpellier, Nice, Toulon, Nimes or Grenoble, to the Institute of Biomedical Research of the French Army (IRBA) in Marseille within less than 72 hours of collection. The Case Report Form was provided at the same time as a paper copy or electronically.

Thin blood smears were stained using a RAL^® ^kit (Réactifs RAL, Paris, France) and examined to determine the *P. falciparum *density and to confirm monoinfection. Parasitized erythrocytes were washed three times in RPMI 1640 medium (Invitrogen, Paisley, UK), buffered with 25 mM HEPES and 25 mM NaHCO_3_. If parasitaemia exceeded 0.8%, infected

erythrocytes were diluted to 0.5-0.8% with uninfected erythrocytes (human blood type A+) and re-suspended in RPMI 1640 medium supplemented with 10% human serum (Abcys S.A. Paris, France) to a haematocrit of 1.5%.

### Drugs

PPQ, PND, DHA and AS were obtained from Shin Poong Pharm Co. (Seoul, Korea). CQ and QN were purchased from Sigma (Saint Louis, MO). MDAQ was obtained from the World Health Organization (Geneva, Switzerland), MQ from Roche (Paris, France) and LMF from Novartis Pharma (Basel, Switzerland). PPQ, QN, MDAQ, MQ, DHA and AS were dissolved first in methanol and then diluted in water to obtain final concentration ranging from 0.8 to 1,000 nM for PPQ, 5 to 3,200 nM for QN, 1.56 to 1,000 nM for MDAQ, 3.2 to 400 nM for MQ and 0.1 to 100 nM for DHA and AS. PND and CQ were dissolved and diluted in water in concentrations ranging between 0.15 to 100 nM for PND, 5 to 3,200 nM for CQ. LMF was dissolved and diluted in ethanol to obtain final concentration ranging from 0.5 to 310 nM.

### Ex vivo assay

The new term '*ex vivo *susceptibility' is used to describe studies on fresh isolates, while the term of '*in vitro *susceptibility' should now refer to studies on strains of parasites, which have been either kept in culture for at least two to three generations or which have been cryo-preserved. For *ex vivo *isotopic microtests, 200 μl/well of the suspension of synchronous parasitized red blood cells (final parasitaemia, 0.5%; final haematocrit, 1.5%) were distributed in 96-well plates pre-dosed with anti-malarial drugs. Parasite growth was assessed by adding 1 μCi of tritiated hypoxanthine with a specific activity of 14.1 Ci/mmol (Perkin-Elmer, Courtaboeuf, France) to each well at time zero. The plates were then incubated for 42 hours in controlled atmospheric conditions that consisted of 10% O_2_, 5% CO_2_, and 85% N_2 _at 37°C with a humidity of 95%. Immediately after incubation, plates were frozen and then thawed to lyse erythrocytes. The content of each well was collected on standard filter microplates (Unifilter GF/B; Perkin-Elmer) and washed using a cell harvester (Filter-Mate Cell Harvester; Perkin-Elmer). Filter microplates were dried, and 25 μl of scintillation cocktail (Microscint O; Perkin-Elmer) was placed in each well. Radioactivity incorporated in nucleotides by the parasites was measured with a scintillation counter (Top Count; Perkin-Elmer).

Internal controls were used (testing of 3D7 and W2 *P. falciparum *clones maintained in continuous culture) in order to validate the results. All strains were synchronized twice with sorbitol before use [16].

Clonality was verified using PCR genotyping of polymorphic genetic markers *msp1*, *msp2*, and microsatellite loci [17,18].

The drug concentration that inhibits 50% of parasite growth (IC_50_) was defined as the drug concentration corresponding to 50% of the incorporation of tritiated hypoxanthine by the parasite in the drug-free control wells. The IC_50 _value was determined by non-linear regression analysis of log-based dose-response curves (Riasmart™, Packard, Meriden, USA).

### Statistical analysis

Data of *ex vivo *susceptibility of *P. falciparum *parasites were analysed after logarithmic transformation and expressed as the geometric mean of the IC_50 _and the 95% confidence interval (95% CI). Cross-resistance between the nine drugs was assessed by a pair-wise correlation of IC_50 _values of all isolates and estimated by coefficient of correlation of Spearman (*rho*) (non-parametric test), coefficient of correlation of Pearson (*r*) (parametric test) and coefficient of determination (*r^2^*).

## Results

**Figure 1:**
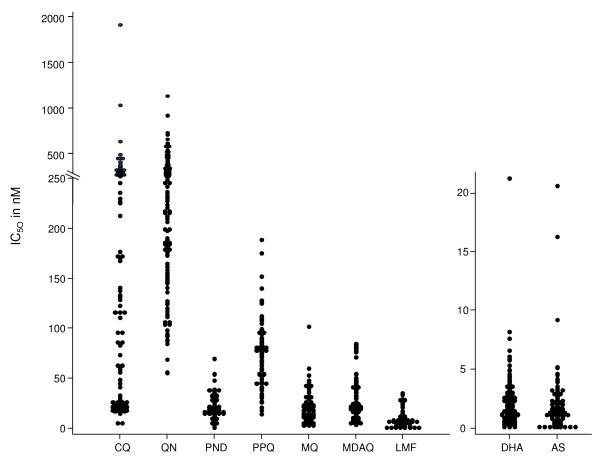
**Dot plots of IC_50 _values in nM for chloroquine (CQ), quinine (QN), pyronaridine (PND), piperaquine (PPQ), mefloquine (MQ), monodesethylamodiaquine (MDAQ), lumefantrine (LMF), dihydroartemisinin (DHA) and artesunate (AS). Each dot represents one isolate**.

One hundred and eighty one *P. falciparum *isolates were tested for their *ex vivo *susceptibility to PPQ, PND, CQ, QN, MQ, MDAQ, LMF, DHA and AS. The mean IC_50_s for these nine anti-malarial drugs are presented in Table 1. The IC_50 _values for PND ranged from 0.55 to 80.0 nM (geometric mean = 19.9 nM, 95% = CI 18.0-022.0) and from 11.8 to 217.3 nM for PPQ (geometric mean = 66.8 nM, 95% CI = 61.8-72.3). The distributions of the IC_50 _for the nine anti-malarial drugs are presented in Figure 1.

**Table 1:** *Ex vivo *susceptibility of the 181 isolates of *Plasmodium falciparum *to pyronaridine (PND), piperaquine (PPQ), chloroquine (CQ), quinine (QN), mefloquine (MQ), monodesethylamodiaquine (MDAQ), lumefantrine (LMF), dihydroartemisinin (DHA) and artesunate (AS)

		IC_50 _in nM						
	
Drugs	No	geometric mean	95% Confidence Interval	min	Q25	median	Q75	max
PND	176	19.9	18.0-22.0	0.55	15.0	19.4	32.1	80.0
PPQ	179	66.8	61.8-72.3	11.8	48.0	76.7	90.5	217.3
CQ	181	79.8	66.6-95.6	5.0	22.9	96.0	246.0	1918.0
QN	181	254.6	234.7-276.1	54.8	182.0	265.0	358.0	1131.0
MQ	181	15.5	13.6-17.6	3.0	8.9	15.8	29.7	166.0
MDAQ	181	22.1	19.1-25.6	1.5	11.8	22.6	43.3	240.0
LMF	181	7.3	6.1-8.8	0.25	5.5	8.0	17.7	114.0
DHA	181	1.3	1.2-1.5	0.10	0.9	1.4	2.4	21.2
AS	179	1.1	1.0-1.3	0.10	0.7	1.3	2.1	20.6

Min: IC_50 _minimum Max: IC_50 _maximun

Q25: 1^st ^quartile Q75: 3^rd ^quartile

*In vitro *cross-resistance was measured by pair-wise correlation of IC_50 _values of 181 isolates (Table 2). A significant positive correlation was shown between responses to PPQ and PND responses (*rho *= 0.46; *P *< 0.00001) and between PPQ and MDAQ (*rho *= 0.30; *P *= 0.0001). No significant correlation was shown between PPQ IC_50 _and responses to other anti-malarial drugs. A significant positive correlation was shown between responses to PND and MDAQ (*rho *= 0.37; *P *< 0.00001), LMF (*rho *= 0.28; *P *= 0.0002), QN (*rho *= 0.24; *P *= 0.0012), AS (*rho *= 0.19; *P *= 0.0108), DHA (*rho *= 0.18; *P *= 0.0173) and CQ (*rho *= 0.16; *P *= 0.0365). The highest coefficients of correlation were shown for correlation between responses to DHA and AS (*rho *= 0.84; *P *< 0.00001), LMF and MQ (*rho *= 0.58; *P *< 0.00001), CQ and MDAQ (*rho *= 0.51; *P *< 0.00001), LMF and DHA (*rho *= 0.46; *P *< 0.00001), LMF and AS (*rho *= 0.45; *P *< 0.00001) or CQ and QN (*rho *= 0.41; *P *< 0.00001).

**Table 2:** Spearman correlation (rho) and Pearson correlation (r^2^) of *in vitro *responses of 181 isolates of *Plasmodium falciparum *to pyronaridine (PND), piperaquine (PPQ), chloroquine (CQ), quinine (QN), mefloquine (MQ), monodesethylamodiaquine (MDAQ), lumefantrine (LMF), dihydroartemisinin (DHA) and artesunate (AS)

		CQ	QN	MQ	DHA	AS	MDAQ	LMF	PND
QN	Rho	0,4143							
	p-value	< 0,00001							
	r^2^	0.159							
MQ	Rho	-0,1254	0,1139						
	p-value	0,0936	0,128						
	r^2^	0.020	0.008						
DHA	Rho	-0,1471	0,134	0,3164					
	p-value	0,0488	0,0729	< 0,00001					
	r^2^	0.026	0.020	0.109					
AS	Rho	-0,1196	0,1291	0,364	0,8374				
	p-value	0,1109	0,0851	< 0,00001	< 0,00001				
	r^2^	0.013	0.013	0.124	0.681				
MDAQ	Rho	0,5071	0,0997	-0,115	-0,1615	-0,0769			
	p-value	< 0,00001 0,1831	0,1243	0,0303	0,3074				
	r^2^	0.227	0.012	0.014	0.028	0.005			
LMF	Rho	-0,1799	0,126	0,5821	0,4636	0,4533	-0,1451		
	p-value	0,0156	0,0918	< 0,00001	< 0,00001	< 0,00001	0,0519		
	r^2^	0.030	0.014	0.300	0.189	0.163	0.012		
PND	Rho	0,1582	0,2425	0,0711	0,1798	0,1933	0,3658	0,2776	
	p-value	0,0365	0,0012	0,3496	0,0173	0,0108	< 0,00001	0,0002	
	r^2^	0.017	0.060	0.003	0.027	0.038	0.101	0.082	
PPQ	Rho	0,036	0,0478	-0,0203	-0,0766	-0,06	0,2988	0,1234	0,4672
	p-value	0,6336	0,5263	0,7881	0,3094	0,4288	0,0001	0,1007	< 0,00001
	r^2^	< 0.001	0.002	< 0.001	0.001	0.001	0.096	0.029	0.230

Isolates with high IC_50 _to at least one of the anti-malarial drug are listed in Table 3. One originated from Thailand with IC_50 _= 71.5 nM for PND associated with IC50 = 91.1 nM for PPQ, IC50 = 1,131 nM for QN, IC50 = 166 nM for MQ, IC50 = 114 nM for LMF, IC50 = 21.2 nM for DHA and IC50 = 16.3 nM for AS.

**Table 3:** Isolates with high IC_50 _to at least one anti-malarial drug among the 181 isolates tested

Isolates	IC_50 _in nM								
origin	PND	PPQ	CQ	QN	MQ	MDAQ	LMF	DHA	AS
Mali	ND	**175**	22	185	23.4	44.8	25.8	1.2	2.0
Comoros	ND	**177.5**	130	460	27.6	76.4	15.8	1.2	3.2
Cameroon	15.0	**189**	171	217	8.0	16.3	7.0	2.0	1.9
Africa	28.4	**217.3**	**1029**	580	31.3	**240.0**	17.7	1.6	1.3
Côte d'Ivoire	31.2	**172.8**	60	377	**68.0**	4.6	58.0	5.3	4.0
Comoros	34.2	83.8	108	**707**	4.4	40.5	20.7	3.3	2.9
Gabon	37.2	90.2	**1918**	580	**40.7**	42.8	25.1	3.4	2.9
Comoros	36.1	**214**	145	292	15.8	69.3	8.1	1.1	1.4
Cameroon	40.2	**181.6**	444	576	3.2	**171**	5.4	0.8	0.6
Comoros	**66.0**	60.2	304	176	18.5	78.9	13.1	3.5	4.6
Niger	**66.3**	79.9	331	393	37.4	61.0	20.1	0.7	0.5
Comoros	**66.7**	82.6	244	**918**	3.5	46.6	14.1	0.8	0.8
Benin	**67.4**	82.4	17	**728**	5.3	21.7	36.4	3.0	2.2
Comoros	**69.4**	46.0	246	210	5.3	82.4	6.4	1.9	1.9
Thailand	**71.5**	91.1	63	**1131**	**166**	34.4	**114.0**	**21.2**	**16.3**
Côte d'Ivoire	**76.1**	91.0	288	660	7.8	56.0	1.4	1.3	1.8
Comoros	**80.0**	82.0	321	587	32.9	47.2	6.7	2.5	3.5

High IC_50_s are in bold

ND: not determined

## Discussion

*Ex vivo *analysis of the susceptibility of *P. falciparum *isolates to anti-malarial drugs has three important attributes. This approach allows firstly to assay the response of clinical isolates to individual drugs that are unmodified by important host factors that influence drug efficacy in vivo. Secondly, the progressive decline in drug susceptibility of isolates from the same site may identify incipient resistance in the parasite population. Finally, strains with reduced anti-malarial susceptibilities can then be established in continuous culture to provide the material needed to investigate novel molecular mechanisms of resistance as well as for tests of susceptibility to other anti-malarial agents.

The continued spread of *P. falciparum *drug resistance to monotherapies has forced a shift toward the use of ACT. Nevertheless, resistance to at least one component of many of the different formulations of ACT currently in clinical use has been documented, and it is feared that the widespread use of ACT will gradually reduce its clinical efficacy. Clinical failures or at least longer parasite clearance times have been described in Cambodia [12]. In addition, prior therapy by amodiaquine-containing ACT selected reduced response to monodesethylamodiaquine, suggested that amodiaquine-containing regimens may rapidly lose efficacy in Africa [15].

One hundred and eighty one *P. falciparum *strains were tested for their *in vitro *susceptibility to PPQ, PND, CQ, QN, MQ, MDAQ, LMF, DHA and AS. The IC_50 _values for PND ranged from 0.55 to 80.0 nM (geometric mean = 19.9 nM, 95% CI = 18.0-22.0). These values are in accordance with previous studies on *P. falciparum *strains (1.9-47.8 nM and 15-49 nM, respectively) [19,20] or in isolates from patients in Thailand cured with PND (geometric mean = 15.7 nM) or that recrudesced after PND treatment (geometric mean = 23.0 nM) [21] but higher than those found in isolates from Cameroon (geometric mean = 3.58 nM), Senegal (geometric mean = 3.8 nM and 4.52 nM), Gabon (geometric mean = 3.0 nM and 1.87 nM) [22-26] and in Indonesia (geometric mean = 1.92 nM) [27]. In addition, PND is also effective *in vitro *against *Plasmodium vivax *isolates (geometric mean = 2.58 nM) [27].

Antagonistic *in vitro *drug interactions between PND and artemisinin derivatives have been described [22,28,29]. Previous studies have demonstrated *in vitro *cross-resistance between PND and DHA or CQ, with coefficients of determination (*r*^2^) of 0.84 and 0.19-0.46, respectively [19,21,23-25]. A low *r^2 ^*(0.20) was determined between PND and AS in *P. falciparum *strains [20]. In the present study, a positive correlation was shown between PND and DHA or AS responses with a coefficient of correlation *rho *= 0.18 and 0.19, respectively. To suggest common mechanisms of action or resistance for two compounds, that could induce cross-resistance, the coefficient of determination must be high, such as the one for AS and DHA (*rho *= 0.84 and *r*^2 ^= 0.68) corresponding to 68% of the variation in the response to DHA is explained by variation in the response to AS. A coefficient of determination of 0.027 or 0.038 means that only 2.7% and 3.8% of the variation in the response to PND is explained by variation in the response to DHA and AS. These data suggest that there is no cross-resistance between PND and artemisinin derivatives. In addition, the combination of PND and AS has undergone successful clinical evaluation in Africa [10,30]. In the present study, a positive correlation was shown between PND and CQ responses with a coefficient of correlation *rho *= 0.16 and *r^2 ^*= 0.017. This means that only 1.7% of the variation in the response to PND is explained by variation in the response to CQ. These data are consistent with the efficacy of the combination of PND and AS for areas in which parasites are resistant to CQ [10,30]. There have been conflicting reports on the correlations between *P. falciparum *responses to PND and CQ. Previous studies showed weak (from 0.003 to 0.26) [20,23-25,27] to modest (0.40 and 0.46) [19,21] coefficients of determination for correlations between PND and CQ. PND appeared to be equally effective *in vitro *against 37 isolates from two areas of Thailand with different CQ resistance levels [31]. Similarly, Basco and Le Bras showed no correlation between resistance to PND and CQ for 31 isolates from Central and West Africa [32]. These results suggest that no cross-resistance exists between PND and CQ. In addition, an isolate collected in a patient who took part in trekking along the Mekong from the south of Laos to the north of Thailand showed high susceptibility to CQ and MDAQ and very low susceptibility to PND (71.5 nM), PPQ (91.1 nM), QN (1,131 nM), MQ (166 nM), LMF (114 nM) and to the artemisinin derivatives DHA (21.2 nM) and AS (16.3 nM) [33]. This multi-resistant isolate was suspected of being resistant to ACT. All of the other significant positive correlations between PND and QN, MDAQ or LMF are too low (*rho *< 0.37, *r*^2 ^< 0.10) to suggest cross-resistance between PND and quinoline drugs. This absence of cross-resistance may be explained by the absence of association between PND and genes involved in quinoline resistance, such as *pfcrt*, *pfmdr1*, *pfmrp *or *pfnhe-1 *[20].

The highest coefficient of correlation was observed between PND and PPQ (*rho *= 0.47 and *r*^2 ^= 0.23). This means that 23% of the variation in the response to PND is explained by variation in the response to PPQ. This result is also too low to suggest cross-resistance between the two drugs. This result was in accordance with previous data (*r*^2 ^= 0.20) [27].

*In vitro *drug interaction between PPQ and artemisinin derivatives was indifferent or antagonistic [34-37]. However, PPQ has been combined with DHA and it has undergone successful clinical evaluation in Africa, Asia and South America [9,38-40]. In addition, PPQ-DHA is also effective to treat *P. vivax *malaria [41]. But unfortunately clinical failures to PPQ have been reported in areas of China where it has been deployed in monotherapy for *P. falciparum *[42].

The IC_50 _values for PPQ ranged from 11.8 to 217.3 nM (geometric mean = 66.8 nM, CI95% 61.8-72.3). These data are in accordance with previous studies on *P. falciparum *strains [35,43] or isolates from Cameroon (geometric mean = 39 nM) [44], Thai-Burmese border (49 nM) [45] and Kenya (50 nM) [46], but superior to geometric mean of isolates from Uganda (6.1 nM) [47], Indonesia (21.8 nM) [48] or Papua New Guinea [49]. The isolate with the highest IC_50 _to PPQ (217.3 nM) was also resistant to CQ (1029 nM) and MDAQ (240 nM).

Encouragingly, PPQ has not shown any correlation with the other quinoline drugs, i.e. CQ, QN, MDAQ, LMF or MQ. These results suggest that no cross-resistance exists between PPQ and CQ and the other quinoline anti-malarial drugs. These data are in accordance with previous studies, which showed weak coefficients of determination included between 0.015 and 0.14 for correlation between PPQ and CQ [43,45-47]. No significant correlation was identified between PPQ and DHA or AS. These data are in accordance with previous results [47]. PPQ has shown no cross-resistance with any of the anti-malarial drug tested. The absence of cross-resistance may be explained by the absence of association between PPQ and genes involved in quinoline resistance, such as *pfcrt*, *pfmdr1*, *pfmrp *or *pfnhe-1 *[43]. A copy number variation event on chromosome 5 could be associated with PPQ resistance [50]. Transgene expression studies are underway with individual genes in this segment to evaluate their contribution to PPQ resistance.

DHA-PPQ is an inexpensive, safe and highly effective treatment for uncomplicated *falciparum *and *vivax *malaria [41,51,52]. DHA-PPQ seems to offer a better post-treatment prophylactic effect following therapy compared with artemether-LMF [6,53,54] or AS-amodiaquine [55]. The significant lower risk of recurrent parasitaemia after treatment with DHA-PPQ is likely to be explained by differences in pharmacokinetics of the non-artemisinin partner drugs. PPQ, a bisquinoline, is estimated to have elimination half-life of 17-33 days [38,56,57] while LMF and MDAQ have an estimated elimination half-life of two to six days and one-10 days [58], respectively.

The PND-AS combination (Pyramax^®^) is one of the latest ACT combinations currently under development by the not-for-profit organisation Medicines for Malaria Venture (Geneva, Switzerland) and the pharmaceutical company Shin Poong Pharmaceuticals (Seoul, Republic of Korea) for the treatment of uncomplicated *P. falciparum *malaria and for the blood stages of *P. vivax *malaria. Pyramax^® ^has recently completed phase III trials in humans. A five-day regimen of PND alone (total dose = 1800 mg) produced a better cure rate than AS, artemether or MQ used alone in the same conditions in Thailand [21]. Efficacy of PND-AS was non-inferior to that of artemether-LMF for treatment of uncomplicated *falciparum *malaria in Africa and Southeast Asia [10].

## Conclusions

In this study, the excellent *in vitro *anti-malarial activities of the ACT components PPQ and PND on *P. falciparum *were confirmed. The absence of cross-resistance with quinolines and artemisinin derivatives confirmed the relevant use of the combinations of PPQ and DHA or PND and AS in areas where parasites are resistant to conventional anti-malarial drugs.

## Competing interests

The authors declare that they have no competing interests. The conclusions presented in this article were not financially influenced.

## Authors' contributions

AP, CR and BP conceived, designed, carried out *in vitro *testing of drug susceptibility, analysed the data and drafted the manuscript. PP, FBV, FS, DM, SP, PD, DB, DM, BF, GM and NB carried out diagnostic, monitoring patients, collected clinical and epidemiological data. CO and ED conceived, designed and drafted the manuscript. All authors read and approved the final manuscript.
